# Pregnancy-induced hypertension and infant growth at 28 and 42 days postpartum

**DOI:** 10.1186/1471-2393-5-10

**Published:** 2005-05-20

**Authors:** Emmanuelle Baulon, William D Fraser, Bruno Piedboeuf, Pierre Buekens, Xu Xiong

**Affiliations:** 1 Department of Obstetrics and Gynecology, CHU Strasbourg, France; 2 Department of Obstetrics and Gynecology, University of Montreal, Quebec, Canada; 3 Department of Pediatrics, Laval University, Quebec, Canada; 4 Department of Epidemiology, Tulane University, New Orleans, LA, USA

## Abstract

**Background:**

No previous studies have examined the effect of pregnancy-induced hypertension (PIH) on early infant growth. The objective was to study infant growth patterns of babies born to mothers with PIH at 28 and 42 days postpartum.

**Methods: Design:**

We conducted a population-based retrospective cohort study of 16,936 pregnancies delivered between January 1, 1989 through December 31, 1990 in Suzhou, China. PIH was classified as gestational hypertension, preeclampsia and severe preeclampsia. Infant Growth Percentage (IGP) was calculated as the weight gain from birth to infant weight at 28 or 42 days postpartum divided by the birth weight. Univariate analysis and multivariate linear regression were performed to compare the infant weight as well as IGP at 28 and 42 days postpartum between various types of PIH and the normotensive group.

**Results:**

Infant weights at 28 and 42 days postpartum were significantly lower in severe preeclampsia (e.g., 4679.9 g at 42 days) and preeclampsia (e.g., 4763.8 g at 42 days) groups than in the normotensive group (e.g., 4869.1 g at 42 days, p < 0.01). However, there were no differences in IGP between groups. After stratifying by intrauterine growth restriction (IUGR) status, if babies were not intrauterine growth restricted, none of the PIH types showed a significantly lower weight at 28 and 42 days postpartum and their IGPs were similar to those of the reference group. When babies were growth restricted, all PIH groups showed significantly lower weights but higher IGP at 28 and 42 days postpartum as compared to the normotensive group.

**Conclusion:**

Infants born to mothers with PIH but without IUGR have normal early infant growth. IUGR secondary to PIH is associated with significant catch-up growth at 28 and 42 days postpartum.

## Background

Pregnancy-induced hypertension (PIH), especially preeclampsia, is a major cause of maternal and perinatal morbidity and mortality worldwide [1,2]. The impact of PIH on birth outcomes has been extensively studied. However, the potential long-term effect of PIH on infants born to PIH mothers has been less studied. PIH has been confirmed to increase significantly the risk of low birth weight by both increasing preterm birth as well as reducing fetal growth. On the other hand, PIH has been found to be associated with an increased rate of high birth weight and large-for-gestational age babies [3,4]. These findings suggest that PIH, more specifically preeclampsia, is a heterogeneous syndrome and that preeclampsia may appear in two forms: restricted fetal growth preeclampsia and normal fetal growth preeclampsia [4-7]. PIH may have different short and long term effects on infant growth between these possible two types of preeclampsia by intrauterine growth restriction (IUGR) [5,6]. Low birth weight or IUGR babies have been associated with the occurrence of several chronic diseases such as cardiovascular diseases in later life [8-10]. However, the hypothesis of the fetal origins of adult disease is still the subject of debate [11,12]. One argument is that maternal risk factors in pregnancy (such as PIH) and environmental risk factors in the postpartum period can contribute to this life-long development of the chronic disorders. A change in infant growth of the IUGR baby itself (e.g. catch-up growth) such as in the critical early infant period may also have long-term effects on health later in life, and this change of postpartum growth may be influenced by PIH. Therefore, it is important to study postpartum infant growth patterns of babies born to mothers with PIH, and to determine if there are differences in infant growth between babies with and those without IUGR. However, to date, there have been few studies on the effects of PIH on infant growth. The objective of this study was to examine the effects of the various types of PIH on infant weight gain at 28 and 42 days postpartum.

## Methods

### Database

We conducted a retrospective cohort study based on a population-based perinatal database from Suzhou, China. The database was previously described by Xiong et al [3,13]. In brief, data used in this analysis were population-based, collected in all 10 hospitals in the city, including16,936 pregnant women from January 1, 1989 through December 31, 1990. We excluded patients with multiple pregnancies (212 cases), chronic cardiovascular disease (36 cases), chronic renal disease or a history of renal disease (303 cases), chronic hypertension or history of hypertension (59 cases), and abnormal blood pressure (diastolic pressure ≥ 90 mmHg or systolic pressure ≥ 130 mmHg) at the first prenatal visit before 21 weeks (850 cases) and abnormal blood pressure after 42 postpartum days (988 cases). There were 2,567 women with no or missing information on infant weight at 28 days or 42 days postpartum, including 105 women who had stillbirths, 138 women who had infant deaths up to 28 days postpartum and 2,324 women who were lost to follow-up. After exclusions, a total of 11,921 pregnancies were retained for the analysis.

### Definition of pregnancy-induced hypertension

PIH was classified as gestational hypertension, preeclampsia, severe preeclampsia or eclampsia. According to the Chinese criteria [14], gestational hypertension was defined as a blood pressure equal to or greater than 130/90 mmHg on more than two occasions greater than six hours apart without proteinuria after 21 weeks of gestation. Preeclampsia was diagnosed as hypertension of equal or greater than 130/90 mmHg but less than 160/110 mmHg with proteinuria of 1+ or 2+ on dipstick in two samples 6 hours apart or greater than 0.3 grams in a 24-hour urine collection. Severe preeclampsia was diagnosed when preeclampsia was complicated by a systolic pressure of ≥ 160 mmHg or diastolic pressure ≥ 110 mmHg and/or if proteinuria was greater than 2+ on dipstick or 5 grams in 24-hour urine collection. Eclampsia was defined as seizure occurred in patients with preeclampsia. Patients with eclampsia were grouped with the severe preeclampsia group for analysis. We restricted our focus to PIH, therefore, pregnancies complicated by chronic hypertension and preeclampsia superimposed on chronic hypertension were not studied.

### Definition of outcomes and confounding variables

Birth weight was measured in grams at birth. Infant weight was measured at 28 and 42 days postpartum. Gestational age was determined by the obstetricians on the basis of the information on menstrual history, physical examination or early ultrasound examination. IUGR was defined as birth weight below the tenth percentile of expected weight for gestational age [15]. Infant weight at 28 days was measured as the standardised medical procedures during the third postpartum home visit by personnel from the community clinic near the newborn's home. Infant weight at 42 days postpartum was measured in hospital during the last day of perinatal care examination while registering for the beginning of the infant care. We developed an index of Infant Growth Percentage (IGP) to measure the infant growth rate. IGP at 28 days is defined as [(infant weight 28 days - birth weight) / birth weight] × 100% and so is the IGP at 42 days by [(infant weight 42 days - birth weight) / birth weight] × 100%. Infant complications were defined as at least one of respiratory distress, newborn fever, birth injury, jaundice, sclerema, cord infection and intra-cranial bleeding. Other potential confounding variables include maternal age, body mass index [weight (kg)/height(m)^2^], infant's sex, pre-pregnancy or gestational diabetes mellitus and maternal anemia.

### Statistical analysis

Mean birth weight and infant weights at 28 and 42 days postpartum were compared by a variance of analysis between different PIH groups. First, the three groups with PIH disorders were compared to the normotensive referent group. Then each PIH group was further divided into two subgroups according to the existence of IUGR and the same analysis performed using normotensive pregnancies without IUGR as the referent group. Post hoc pair-wise multiple comparisons, which test which specific means differ significantly from others, were performed by Tukey and Bonferroni procedures [16]. In order to assess the independent effect of PIH on infant weight at 28 and 42 days postpartum respectively, multiple linear regression analysis was performed to adjust for confounding variables [16,17]. Infant weight at 28 days and 42 days were the dependent variables. Dummy variables of severe preeclampsia with and without IUGR, preeclampsia with and without IUGR, gestational hypertension with and without IUGR, normotensive status with and without IUGR, and other confounding variables were independent variables. The regression coefficients (β) are estimated by the method of least squares [16]. The statistical significance (p-value) of β was also tested. All statistical analyses were performed with SPSS 10.0 for Windows (SPSS Inc., Chicago, IL).

## Results

Table 1 summarizes the demographic and reproductive characteristics of the women classified in each of the categories of the three PIH groups. Among the11,921 pregnant women, PIH occurred in 1,252 women (10.5 %). Mean maternal age at delivery was 25.8 years, 95.3 % of women were nulliparous. Almost all women gave birth at hospital (99.8 %). Mean (± SD) gestational age at delivery was 39.30 weeks (± 1.55 weeks), with a shorter gestational age for severe preeclampsia. The incidences of preterm birth (<37 weeks of gestation) and low birth weight (<2,500 g) were significantly higher among babies born to mothers with preeclampsia and severe preeclampsia than normotensive women. Infant complications occurred in 7 % of babies and were more frequent in preeclampsia and severe preeclampsia groups.

**Table 1:** Demographic and reproductive characteristics of study population by pregnancy-induced hypertension, Suzhou, China, 1989–1990

	Subjects						
						
Characteristics*	No.	%	Normotensive (N=10,669) %	Gestational hypertension (N=782) %	Preeclampsia (N=365) %	Severe preeclampsia (N=105) %	Statistical significance (χ^2^test)
Maternal age							
≤30	11,230	94.2	94.6	90.3	91.5	94.3	0.000
> 30	691	5.8	5.4	9.7	8.5	5.7	
							
Parity							
Nulliparous	11,365	95.3	95.3	94.9	97.8	97.1	0.100
Multiparous	556	4.7	4.7	5.1	2.2	2.9	
							
Sex							
Male	6,026	50.5	50.8	48.6	47.4	55.2	0.290
Female	5,895	49.5	49.2	51.4	52.6	44.8	
							
Preterm birth (<37 weeks)							
No	11,527	96.7	96.6	98.2	96.7	93.3	0.023
Yes	394	3.3	3.4	1.8	3.3	6.7	
							
Low birth weight (<2,500 g)							
No	11,620	97.5	97.6	97.3	95.1	92.4	0.000
Yes	301	2.5	2.4	2.7	4.9	7.6	
							
Mean gestational age (Weeks)	39.30		39.29	39.44	39.30	38.69	0.000 (Analysis of variance)
Place of delivery							
Hospital	11,900	99.8	99.8	99.7	100	100	0.981
Home	11	0.1	0.1	0.1	0	0	
							
BMI							
< 24	11,012	92.8	93.1	90.0	89.6	93.3	0.005
24–28	426	3.6	3.4	5.3	5.8	4.8	
> 28	432	3.6	3.5	4.7	4.6	1.9	
							
Infant complications No Yes							
No	10,989	93.1	93.1	90.9	87.0	88.5	0.000
Yes	810	6.9	6.4	9.1	13.0	11.5	

* Excluding cases with missing information.

Table 2 presents the overall effects of PIH on infant growth at 28 and 42 days postpartum. The infant weights were statistically significantly lower in infants born to mothers with both preeclampsia and severe preeclampsia than infants born to normotensive mothers. However, IGPs were not different between groups. For example, at 42 days postpartum, weights were significantly lower for the preeclampsia and severe preeclampsia groups than for normotensive group (mean weight = 4,763 g and 4,679 g vs. 4,869 g, p < 0.001), but no evidence of catch-up growth in infants born to mothers with preeclampsia and severe preeclampsia (IGP = 47.7% and 48.3% vs. 49.7%, p = 0.618).

**Table 2:** Pregnancy-induced hypertension and infant weight at 28 and 42 days postpartum, univariate analysis

			Infant growth 28 days postpartum			Infant growth 42 days postpartum		
			
	Mean birth weight (g)	SD†	Infant weight (g)	SD	Infant growth percent ^1 ^(%)	Infant weight (g)	SD	Infant growth percent ^2 ^(%)
Normotensive (N=10,669)	3252.4 (Referent)	416,1	4088.8 (Referent)	496.2	25.7	4869.1 (Referent)	609.9	49.7
GH# (N=782)	3288.8	448.9	4084.1	540.4	24.2	4878.6	696.2	48.3
PE¶ (N=365)	3224.9	465.8	4018.6*	513.7	24.6	4763.8**	631.8	47.7
Severe PE (N=105)	3170.9	502.9	3941.9*	564.9	24.3	4679.9**	727.3	47.6
Total (N=11,921)	3253.2	420.9	4085.0	500.7	25.6	4864.8	618.2	49.5

* p < 0.05, ** p < 0.01

^1 ^[(Infant weight 28 days - birth weight) / birth weight] × 100 %, ^2 ^[(Infant weight 42 days - birth weight) / birth weight] × 100 %

† SD: standard deviation, #GH: gestational hypertension, ¶PE: preeclampsia.

Table 3 presents the effect of PIH on growth at 28 and 42 days postpartum, stratified for the presence or absence of intrauterine growth restriction (IUGR). Infant weights were not different between infants born to mothers with any types of PIH *without IUGR *and normotensive babies without IUGR, and IGPs were not different between any types of PIH and non-IUGR normotensive control. Conversely, infant weights were markedly lower in all infants *with IUGR *than normotensive controls without IUGR. Infant weight gains and IGPs were higher in any types of PIH and normotensive control *with IUGR *than in their counterpart groups *without IUGR*. For example, at 42 days postpartum, *in the absence of IUGR*, there were no differences in infant weight between any of the types of PIH (ranging from 4,801 g to 4,929 g) and non-IUGR normotensive group (4,905 g). The IGPs were not different between any types of PIH (ranging from 46.2 % to 47.0 %) and non-IUGR normotensive control (48.6%). However, *in the presence of IUGR*, infant weights were markedly lower in the three types of PIH (ranging from 3,882 g to 4,231 g) and in normotensive (4,255 g) groups than in non-IUGR normotensive control group (4,905 g), p < 0.001. The IGPs were significantly higher (ranging from 59.8% to 71.3%) as compared to the reference group (48.8%), suggesting a significant catch-up growth.

**Table 3:** Pregnancy-induced hypertension and infant weight at 28 and 42 days postpartum by newborn IUGR status, univariate analysis

		Infant growth 28 days postpartum			Infant growth 42 days postpartum		
		
	Mean birth weight (SD†) (g)	Infant weight (SD) (g)	Weight gain (g)	Infant growth percent ^1 ^(%)	Infant weight (SD) (g)	Weight gain (g)	Infant growth percent ^2 ^(%)
Normotensive / non-IUGR‡ (N=10,067)	3296.6 (382.0) (Referent)	4126.9 (471.3) (Referent)	830.3	25.2 (Referent)	4905.8 (592.9) (Referent)	1609.2	48.8 (Referent)
Normotensive / IUGR (N=602)	2513.2 (219.9)**	3451.1 (466.9) **	937.9	37.3**	4255.8 (563.3)**	1742.6	69.3**
GH# / non-IUGR (N=725)	3353.1 (395.4)**	4143.6 (500.4)	790.5	23.6	4929.4 (665.8)	1576.3	47.0
GH / IUGR (N=57)	2469.8 (230.9)**	3327.4 (454.8)**	857.5	34.7**	4231.6 (754.3)**	1761.8	71.3**
PE¶/ non-IUGR (N=326)	3314.9 (401.8)	4091.3 (463.8)	776.4	23.4	4853.1 (572.9)	1537.2	46.4
PE / IUGR (N=39)	2472.1 (215.6)**	3410.4 (512.9)**	938.3	37.9**	4025.4 (624.2)**	1553.3	62.8**
Severe PE / non-IUGR (N=91)	3285.2 (434.8)	4037.4 (518.9)	752.2	22.9	4801.8 (682.8)	1516.6	46.2
Severe PE / IUGR (N=14)	2428.6 (171.8) **	3321.4 (459.0) **	892.8	36.8**	3882.1 (463.5)**	1453.6	59.8
Total (N=11,921)	3253.2 (420.9)	4085.0 (500.7)	831.8	25.6	4864.8 (618.2)	1611.6	49.5

* p < 0.05, ** p < 0.01

^1 ^[(Infant weight 28 days - birth weight) / birth weight] × 100 %, ^2 ^[(Infant weight 42 days - birth weight) / birth weight] × 100 %

#GH: gestational hypertension, ¶PE: preeclampsia, ‡ IUGR: intrauterine growth restriction.

† SD: standard deviation

Table 4 presents mean birth weight differences, infant weight differences at 28 days and 42 days postpartum between various types of PIH with and without IUGR and the normotensive control group without IUGR, as well as the corresponding regression coefficients (i.e., the weight differences after adjustment for confounders). The results of multivariate linear regression were consistent with those of univariate analysis (Table 3). After adjustment for confounding variables, IUGR babies born to any of the PIH groups had significantly lower infant weights at 28 and 42 days postpartum. For example, there was a 903 g (1,023 g before the adjustment for confounders) reduction in infant weight at 42 days postpartum in IUGR babies born to severe preeclamptic mothers relative to non-IUGR babies born to mothers with normal blood pressure, although the IGP was higher in the IUGR babies.

**Table 4:** Pregnancy-induced hypertension and infant weight at 28 and 42 days postpartum, multivariate analysis

	Mean birth		Infant weight 28 days postpartum		Infant weight 42 days postpartum	
			
	Mean birth weight difference (g)	β (SE)§	Infant weight difference (g)	β (SE)	Infant weight difference (g)	β (SE)
Normotensive / non-IUGR‡ (N=10,067)	Ref.	Ref.	Ref.	Ref.	Ref.	Ref.
Normotensive / IUGR (N=602)	783.4**	776.2 (15.4)**	675.8**	640.0 (19.4)**	650.0**	619.4 (25.2)**
GH# / non-IUGR (N=725)	-56.6**	-34.0 (14.0)	-16.7	-4.4 (18.6)	-23.7	-12.8 (22.8)
GH / IUGR (N=57)	826.7**	825.4 (49.0)**	799.5**	815.1 (61.8)**	674.2**	678.9 (80.1)**
PE¶/ non-IUGR (N=326)	-18. 4	-6.97 (20.5)	35.6	38.0 (25.8)	53.6	55.4 (33.5)
PE / IUGR (N=39)	824.5**	795.9 (60.3)**	716.5**	654.1 (76.0)**	880.4**	835.2 (98.7)**
Severe PE / non-IUGR (N=91)	11.4	-20.0 (38.8)	89.5	39.4 (48.9)	104.0	69.9 (63.5)
Severe PE / IUGR (N=14)	868.0**	796.8 (92.4)**	805.5	674.7 (116.5)**	1023.6**	903.0 (151.2)**

* p < 0.05; ** p < 0.01

§β, coefficient, SE, standard error; adjusted for maternal age, body mass index, diabetes, infant's sex, maternal anemia, gestational age

#GH: gestational hypertension, ¶PE: preeclampsia, ‡ IUGR: intrauterine growth restriction

## Discussion

To our knowledge, there is no previous study that has examined the effect of PIH on infant growth in the early infant period. Our study found that when babies were born with IUGR, all PIH groups had lower infant weight and significant 'catch-up' growth at 28 and 42 days postpartum. However, if babies were not born with IUGR, any type of PIH did not have detrimental effect on infant growth at 28 and 42 days postpartum. Our study suggests that PIH per se does not affect early infant growth.

The finding of different effects of PIH on early infant growth according to the presence of IUGR further supports the hypothesis that preeclampsia is a heterogeneous disorder and present at least two subtypes: IUGR vs. normal fetal growth preeclampsia [4-6]. These two subtypes of preeclampsia may have different pathogeneses, clinical manifestations and short and long term effects on both infants and mothers [4-7]. Patients with preeclampsia who have IUGR babies may have placental dysfunction such as reduced utero-placental perfusion as the common pathogeneses. The present study shows this group of infants has sustained lower weight in the early infant period. In contrast, patients with preeclampsia who have babies with normal fetal growth may not have the significant placental dysfunction, thus have normal intrauterine as well as early infant growth.

Newborns with poor intrauterine growth often demonstrate postnatal catch-up growth [18,19]. Our study suggests such a catch-up growth is initiated soon after birth. The impact of catch-up growth on infant's long-term health remains unclear. There is a dilemma as to whether this catch-up growth, either inherent to IUGR babies or through growth promotion programs as promoted in many developing countries, is good or bad. In short term, such a catch-up growth enables infants to accelerate growth in order to reach their normal growth curve, resulting in reduced infant morbidity and mortality [19]. However, it has been suggested that catch-up growth could have a detrimental influence, leading to the onset of certain chronic diseases in later life [20-23]. The crucial time for the development of long-term consequences is the early postnatal period when catch-up growth occurs in around 80 % of IUGR babies [18,22]. Some studies [8,12,20,22] found that hypertension, cardiovascular diseases and mellitus diabetes in adult life were correlated with the existence of a low birth weight and a catch-up growth in the early postnatal period. The hypothesis of the fetal origins of adult disease [10,24,25] may be redefined to consider the impact of the early infant period on the risk of adult diseases [22,23]. Our study indicates that for the infants born to mothers with various types of PIH, only those babies with IUGR showed a catch-up growth. Future long-term follow up studies are needed to examine if IUGR babies born to PIH mothers are at higher risk to develop certain adult diseases later in life than those non-IUGR babies born to PIH mothers.

Several limitations of this study have to be addressed. First, our database does not have information on important factors such as maternal smoking and breastfeeding. The prevalence of smoking is high for men in China, however, reported smoking is rare among women (<0.5%) [26]. Therefore, our results are not likely to be biased by not controlling for smoking. To our knowledge, almost all women breast-fed their infants at that time. However, it is possible that the presence of PIH or IUGR affected breastfeeding and thereby early infant growth. Second, the database has many cases with missing information on weight at 28 days and/or 42 days postpartum. We compared these missing cases with those pregnant women remained for analysis. The rate of preterm birth and low birth weight were higher in missing cases than in the remained study population. This is partly due to women who had perinatal deaths up to 28 days postpartum (most of those babies were preterm, IUGR, or low birth weight) and thus had no information on infant weight at 28 and 42 days postpartum were classified into missing cases. This is the reason why the study population has fewer preterm birth, low birth weight and intrauterine restriction infants. However, there were no significant differences in demographic and reproductive characteristics such as pregnancy-induced hypertension, maternal age, parity, number of prenatal visits, infant's sex and gestational age. Third, weight can be a good growth indicator but, ideally, should be correlated to height and head circumference [27], in order to differentiate between symmetrical and non-symmetrical IUGR. We only considered weight as a measure of growth pattern because it was the only available variable.

## Conclusion

The effect of PIH on early infant growth is dependent on whether or not a baby is intrauterine growth restricted. IUGR secondary to PIH is associated with significant catch-up growth in early infant period. Therefore, we speculate that IUGR babies born to mothers with PIH may be at higher risk to develop certain chronic diseases in their later life. More studies in North American or European populations are needed to confirm these results.

## Competing interests

The author(s) declare that they have no competing interests.

## Authors' contributions

Each author has contributed to the concept, the design and the analysis on the study and to the writing of the draft. All authors read and approved the final version.

## Pre-publication history

The pre-publication history for this paper can be accessed here:


